# The association of diet with quality of life, disability, and relapse rate in an international sample of people with multiple sclerosis

**DOI:** 10.1179/1476830514Y.0000000117

**Published:** 2015-04

**Authors:** Emily J Hadgkiss, George A Jelinek, Tracey J Weiland, Naresh G Pereira, Claudia H Marck, Dania M van der Meer

**Affiliations:** 1 Emergency Practice Innovation Centre, St Vincent's Hospital Melbourne, Victoria, Australia; 2 Department of Epidemiology and Preventive Medicine, Monash University, Victoria, Australia; 3 Department of Medicine, The University of Melbourne (St Vincent's Hospital), Victoria, Australia; 4 Faculty of Medicine, Notre Dame University, Fremantle, Western Australia, Australia

**Keywords:** Diet, Disability, Multiple sclerosis, Quality of life, Relapse

## Abstract

**Objectives:**

To explore the association between dietary factors including fat, fruit and vegetable intake, dairy and meat consumption, and health-related quality of life (HRQOL), disability and relapse rate in a large international sample of people with multiple sclerosis (MS).

**Methods:**

Participants with MS were recruited to the study via Web 2.0 platforms and completed a comprehensive survey measuring demographic and clinical characteristics, HRQOL, disability, relapse rate, and the Diet Habits Questionnaire (DHQ).

**Results:**

Of 2469 participants with confirmed MS, 2087 (84.5%) provided complete data on their dietary habits (DHQ total score). Multivariate regression models demonstrated that every 10-point increase on the DHQ total score was associated with nearly a six-point and five-point increase in physical and mental HRQOL, respectively, and 30.0% reduced likelihood of a higher level of disability. After controlling for age and gender, and the other dietary covariates, ‘healthy’ consumption of fruit and vegetables and dietary fat predicted better quality of life and less likelihood of higher disability when compared to respondents with a ‘poor’ diet. For those with relapsing–remitting MS, the DHQ total significantly predicted a lower relapse rate and reduced odds of increasing disease activity, but the model fit was poor and the predicted change only marginal.

**Discussion:**

This study supports significant associations of healthy dietary habits with better physical and mental HRQOL and a lower level of disability. Further research is urgently required to explore these associations including randomized controlled trials of dietary modification for people with MS.

## Introduction

Multiple sclerosis (MS) is a chronic inflammatory and neurodegenerative disease of the central nervous system (CNS), characterized by axonal injury and demyelination. The aetiology of MS is still poorly understood, but immune dysregulation caused by a complex interplay of genetic and environmental risk factors appears central to the disease process.^1^ The incidence of MS varies greatly with geographical distribution and migration in childhood appears to influence risk of the disease developing, suggesting an important role for non-infectious environmental determinants.^2^ There is, however, no evidence of genetic associations in MS clinical course or disease severity;^3^ environmental factors^4^ therefore present important opportunities for secondary and tertiary prevention of MS.

Epidemiological data show a higher prevalence of MS in countries of affluence, further from the equator where high caloric, high fat diets are common.^5^ The association between MS risk and diets high in saturated and animal fats and low in polyunsaturated fats has been observed in multiple ecological studies.^6–9^ Case–control studies also support trends towards higher MS risk for greater saturated fat and meat consumption, while vegetable protein, dietary fibre, and certain micronutrients appear to be protective.^10–12^ By contrast, analyses of diet among MS cases in the prospective Nurses Health Study, interestingly, did not support these findings.^13^ Perhaps the most important study of nutrition and MS, led by Swank, involved a longitudinal follow up of people with MS trialled on a very low saturated fat diet supplemented with cod liver or vegetable oils.^14^ This study was commenced before the era of randomized controlled trials, but the vastly better health of trial participants after 34 years compared to those who did not maintain the diet, provides compelling data about the pivotal role of dietary fat in MS. A more recent randomized controlled trial of a low fat diet supplemented with fish oil demonstrated relapse rate reduction, physical, and mental health improvements.^15^

Emerging evidence suggests dyslipidaemia may be implicated in MS outcomes; patients with vascular comorbidities have a higher risk of disability progression.^16^ Higher total cholesterol and low-density lipoprotein are associated with worsening disability in MS patients^17^ and increased cumulative number of lesions on MRI in high-risk clinically isolated syndrome (CIS) patients,^18^ while high-density lipoprotein is associated with lower lesion volume.^17^ Indeed, it has been proposed that MS is not an autoimmune disease but rather a dysfunction of lipid metabolism, with comparisons drawn with the pathophysiological pathways of atherosclerosis.^19^

The role of molecular mimicry, that is, the structural similarity between myelin autoantigens and dietary proteins, has also been proposed as a potential cause of autoimmunity and myelin breakdown in MS. In particular, proteins of the milk fat globule membrane from cow's milk have been implicated. The main suspect is butyrophilin protein, which is structurally similar and induces antibody cross-reactivity to the myelin oligodendrocyte glycoprotein (MOG)^20,21^ and has been shown to stimulate T-cell responses to MOG and induce CNS inflammation in an animal model.^22^ The results of these experimental immunological studies are in line with early epidemiological data showing a high correlation between cow's milk consumption and MS prevalence.^23,24^

Other nutritional components explored for their potential benefit in MS include antioxidants, such as polyphenols and carotenoids, which are found in abundance in fruits and vegetables. Their role in reducing oxidative stress and subsequently, inflammation and neuronal damage,^25^ affords them a possible protective function in MS. To date, findings are inconclusive, with a prospective study finding no association between fruit and vegetable consumption, vitamin supplementation and risk of MS,^26^ and no sufficient data on disease progression outcomes.

Conventional pharmacological therapies have demonstrated modest efficacy in reducing relapse rates or slowing the progression of the disease, but there remain concerns regarding side effects, adverse events, and cost-effectiveness.^27^ Consequently, many people with MS favour a multifaceted approach to their disease management. Dietary modification has been widely considered and undertaken by many patients with MS and there is a large amount of grey literature exploring dietary intervention: an Internet search of the terms ‘diet’ and ‘multiple sclerosis’ gives rise to more than 27 million hits.^28^ Lifetime use of dietary intervention was reported by 41% of people with MS in a study from Germany.^29^ A survey of people with MS from South Australia found that the most common dietary interventions included low fat diets, followed by sugar-free/low sugar diets; wheat or gluten-free diets; and the MS-specific ‘Swank diet’.^30^ However neither study provided detailed information on the dietary habits of people with MS or correlated these behaviours with validated health-related outcomes. Few interventional studies have been conducted to explore the relationship between dietary factors and MS; a Cochrane review of dietary interventions in MS found only six studies met standards of selection criteria and all were trials of polyunsaturated fatty acid (PUFA) supplementation.^28^

This study is part of a wider research project, the *H*ealth *O*utcomes and *L*ifestyle *I*ntervention in a *S*ample of People with *M*ultiple Sclerosis (HOLISM) study. Other papers published previously from the HOLISM dataset correlated MS health-related outcomes with: fish consumption and omega-3 supplementation; and alcohol and smoking. The aim of this study was to explore the association between dietary factors including fat, fruit and vegetable intake, dairy and meat consumption, and health-related quality of life (HRQOL), disability and relapse rate in a large international sample of people with MS.

## Materials and methods

### Subjects and recruitment procedure

The methodology of the HOLISM study has been described in detail in an earlier paper.^31^ In brief, participants were recruited to this study through interactive online sites specifically for people with MS including websites, Facebook, Twitter, forums, and blogs. Participants were asked to read detailed study information before providing consent and continuing to a comprehensive online survey. In order to be eligible for the study, participants were asked to confirm that they were over 18 years of age and that they had received a formal diagnosis of MS from a medical doctor. Those who could not confirm their diagnosis but had CIS or ‘possible MS’ were still able to complete the questionnaire but were excluded from analysis in this study. Participant contact details were recorded for the purpose of follow up over a 5-year timeframe. St Vincent's Hospital Melbourne Human Research Ethics Committee granted ethical approval (LRR 055/12).

### Data collected and tools used

The online survey was comprehensive, taking around 40 minutes to complete. Specific to this study were items exploring: sociodemographics; body mass index (BMI; World Health Organisation criteria); HRQOL; level of gait disability; doctor-diagnosed relapse rate; and dietary habits. All data were self-reported.

*HRQOL*: The Multiple Sclerosis Quality of Life (MSQOL-54) was used to measure HRQOL.^32^ This instrument was developed from the RAND Health Survey Short Form (SF-36) and consists of 52 items, giving rise to 12 scales physical health composite (PHC) and mental health composite (MHC) scores. The MSQOL-54 has been extensively validated and is widely used across MS literature.

*Disability:* The Patient-Determined Disease Steps (PDDS) is a self-reported surrogate tool to the Expanded Disability Status Scale (EDSS), commonly used by neurologists to assess gait disability.^33,34^ The PDDS is scored ordinally from 0 (normal) to 8 (bed bound). It correlates well with the EDSS^35^ and has been used in studies associated with the North American Research Committee on Multiple Sclerosis (NARCOMS) registry.

*Relapse rate:* Participants were asked the number of relapses they had in the previous 12 months and 5 years, as diagnosed by a medical doctor.

*Dietary habits:* The Diet Habits Questionnaire (DHQ) is a 24-item questionnaire used to assess the dietary habits of an Australian cardiac population.^36^ The tool and scoring were developed in line with the National Heart Foundation of Australia Nutrition Recommendations and the National Health and Medical Research Council and Commonwealth Department of Health and Ageing Dietary Guidelines for Australian Adults and is based on two validated measures, taking into account the type and quality of fat consumed.^37,38^ For example, healthy fruit and vegetable intake would be considered at least five vegetable and two fruit servings per day, the regular consumption of legumes and raw nuts or seeds. A healthy fat intake would include fish consumption, the selection of avocado and mono/polyunsaturated oils for salads and spreads, minimal use of oil in cooking and infrequent consumption of processed or fatty meats, full-fat milk, cakes, biscuits, and takeaway foods, among other items. The questionnaire considers both the type of food consumed and the methods of food preparation. Each item scores from 1 to 5 with a score of 1 indicating very poor dietary behaviour and a score of 5 indicating healthy dietary behaviour. These items give rise to 10 dietary subscores: cereals, fruit and vegetables, omega-3 fatty acids, food choices, food preparation, takeaways and snacks, fat, fibre, sodium, and alcohol (some of which share common items). Mean scores ranging from 1 to 5 are calculated for each subscore. Inter-rater agreement on the DHQ scoring method was moderate to high (kappa 0.77–1.0) for three subscores assessed.^36^ The tool is widely used in a cardiac rehabilitation setting but has not been validated in an MS population. For the purpose of our study, four items were excluded from the survey: three items regarding sodium intake and one item on alcohol was replaced with an alternate alcohol assessment measure. The removal of these items did not affect the calculation of the remaining 8 dietary subscores.

### Data analysis

Data were analysed using IBM SPSS Statistics 21.0. PHC and MHC scores of the MSQOL-54 data were calculated according to the original scoring form. The PDDS was collapsed from nine into three ordinal categories for the purpose of analysis. Throughout this paper, these groups will be referred to as mild (scores 0–2), moderate (3–5), and major disability (6–8).

A 5-year annualized relapse rate was calculated by dividing the 5-year relapse rate by the number of years with disease (with an upper limit of five). A pre-determined variable, ‘Disease Activity’, was categorized as increasing, decreasing, or stable, where relapse rate in the previous 12 months was higher, lower, or the same (respectively) as the 5-year annualized relapse rate.

For the purpose of this study, seven dietary subscores were calculated according to the DHQ scoring instructions: cereals, fruit and vegetables, food choices, food preparation, takeaway and snacks, fibre, and fat. Omega-3 fatty acid consumption and alcohol consumption have been reported in previous papers.^39,40^ A total summary score of 20 DHQ items was calculated, giving equal weight to all items, with possible score range of 20–100 (higher scores indicating a more healthy diet). Total item completion was required to determine subscores or the DHQ total score. In our study, Cronbach's alpha coefficient of the DHQ total score was 0.842, indicating reliable internal consistency (Table 1). In addition to the DHQ total, two subscores were selected for more detailed analysis: the fruit and vegetable subscore (Chronbach's alpha 0.724) and the fat subscore (Chronbach's alpha 0.792). These subscores were collapsed into three categories: poor (score 1 < 3.5); moderate (score 3.5 < 4.5); and healthy (score 4.5–5). Respondents were also grouped based on whether they consumed dairy products (one item) or animal meat (excluding fish, two items).

**Table 1:** Summary of DHQ subscores and total score

	Items*	Chronbach's alpha	*n*	Mean score (95% CI)	Median score (IQR)
Cereals	3	0.551	2264	3.55 (3.51–3.59)	3.67 (3.00–4.33)
Fruit and vegetables	5	0.724	2238	3.61 (3.58–3.65)	3.60 (3.00–4.20)
Food choices	4	0.612	2247	4.02 (3.98–4.06)	4.25 (3.50–4.75)
Food preparation	5	0.510	2226	4.44 (4.41–4.47)	4.70 (4.08–4.93)
Takeaway and snacks	3	0.656	2269	3.99 (3.95–4.03)	4.22 (3.33–4.67)
Fibre	8	0.742	2205	3.59 (3.55–3.62)	3.63 (3.00–4.13)
Fat	13	0.792	2155	4.13 (4.10–4.16)	4.23 (3.69–4.69)
DHQ total score	20	0.842	2087	79.0 (78.5–79.5)	80.3 (70.2–89.0)

*n* is the number of respondents who completed all items to give rise to the subscore or total score.

*Some items overlap across subscores.

Bivariate analyses were first undertaken to explore associations between the total score on the DHQ, two subscores, dairy and meat consumption with four measures of health outcomes: HRQOL and disability for all respondents, and relapse rate and disease activity only for those with relapsing–remitting MS. Continuous data were summarized using mean (95% confidence interval (95% CI) or standard deviation (SD)) and categorical data using number and percentage. Independent samples *t-*test was used to compare two groups on continuous end points, and comparison involving three or more independent groups was conducted with one-way analysis of variance (ANOVA) with Tukey or Games–Howell *post-hoc* analyses (based on assessment of homogeneity of variance). Pearson product-moment correlation was used to assess the strength and direction of the relationship between two continuous variables. Categorical data involving contingency tables with three or more groups were analysed with the Pearson's Chi-square test with adjusted standardized residuals to indicate under- or over-representation of groups with a cut-off set at ±2.0.

Significant findings from bivariate analyses were explored with multivariate models to identify independent predictor variables. Two models were constructed for each health outcome: the first with the DHQ total score, and the second with the fruit and vegetable subscore, fat subscore, meat, and dairy. Both models controlled for age and gender. Multiple regression (enter method) was used to predict HRQOL. Preliminary tests were undertaken to assess independence, normality, linearity, and homoscedasticity as well as checking for any outliers (standardized residuals <−3.0 or >3.0). Variance inflation factor <10 and correlations between predictors <0.7 were used as the criteria for absence of multicollinearity. To predict a higher level of disability, ordinal logistic regression was used. Models were considered valid if they met the proportional odds assumption and the overall −2 log-likelihood Chi-square test. Binary logistic regression (‘Enter’ method) assessed disease activity, predicting increasing (worsening) disease activity over decreasing/stable activity. Goodness of fit was assessed with the Hosmer and Lemeshow test and the omnibus test of model coefficients. Count data for relapse rate were over-dispersed; consequently negative binomial logistic regression was used in preference to Poisson regression. Two-tailed tests of significance were used with significance set at 0.05.

## Results

Of the 2469 participants with confirmed MS, 2087 (84.5%) completed all items in the DHQ, allowing a total summary score to be calculated (‘DHQ total’). These respondents were predominantly female (82.3%), highly educated, with a mean age of 45.5 years and living in 52 different countries – mostly English speaking, higher income nations (Table 2). The majority (61.5%) had a relapsing–remitting type of MS, a mild level of disability (scores 0–2), and the mean time since diagnosis was 8.5 years. Most respondents were in a ‘normal’ BMI range but 41.9% were categorized as overweight or obese. With regards to the DHQ measure, there were 2155 (87.3%) complete responses for the fat subscore and 2238 (90.6%) for the fruit and vegetable subscore (Table 3). As previously reported, 37.8% of respondents did not consume dairy and 26.7% did not consume meat.^31^

**Table 2:** Demographics and clinical characteristics of participants who completed the DHQ

Variable	*n* (%), unless stated otherwise
Age (years), mean (SD)	45.5 (10.6)
Gender	
Female	1697/2063 (82.3)
Male	366/2063 (17.7)
Country of residence	
USA	663/2087 (31.8)
Australia	550/2087 (26.4)
UK	356/2087 (17.1)
New Zealand	178/2087 (8.5)
Canada	92/2087 (4.4)
Other (47 countries)	248/2087 (11.9)
Highest level of education completed	
Secondary school or less	514/2082 (24.7)
Vocational training	328/2082 (15.8)
Bachelor degree	759/2082 (36.5)
Postgraduate degree	481/2082 (23.1)
Years since diagnosis, mean (SD)	8.5 (7.3)
Type of MS	
Relapsing–remitting	1280/2080 (61.5)
Primary progressive	149/2080 (7.2)
Secondary progressive	235/2080 (11.3)
Progressive relapsing	42/2080 (2.0)
Benign	88/2080 (4.2)
Unsure/other	286/2080 (13.8)
Level of disability	
Normal	684/2077 (32.9)
Mild disability	318/2077 (15.3)
Moderate disability	155/2077 (7.5)
Gait disability	328/2077 (15.8)
Early cane	229/2077 (11.0)
Late cane	156/2077 (7.5)
Bilateral support	119/2077 (5.7)
Wheelchair/scooter	85/2077 (4.1)
Bedridden	3/2077 (0.1)
BMI categories	
Underweight	89/2062 (4.3)
Normal	1108/2062 (53.7)
Overweight	481/2062 (23.3)
Obese	384/2062 (18.6)
12-month relapse rate*, mean (SD)	0.71 (1.03)
PHC, mean (SD)	59.6 (21.4)
MHC, mean (SD)	67.0 (21.3)

*Subset of participants with relapsing–remitting type.

**Table 3:** Summary of dietary subgroups used for analysis

Dietary subgroups		Whole sample, *n* (%)	Relapsing–remitting participants, *n* (%)
Fruit and vegetable subscore	Poor (1 < 3.5)	943/2238 (42.1)	576/1368 (42.1)
	Moderate (3.5 < 4.5)	940/2238 (42.0)	581/1368 (42.5)
	Healthy (4.5–5)	355/2238 (15.9)	211/1368 (15.4)
Fat subscore	Poor (1 < 3.5)	388/2155 (18.0)	247/1321 (18.7)
	Moderate (3.5 < 4.5)	984/2155 (45.7)	588/1321 (44.5)
	Healthy (4.5–5)	783/2155 (36.3)	486/1321 (36.8)
Meat consumption	Yes	1681/2292 (73.3)	1003/1399 (71.7)
	No	611/2292 (26.7)	396/1399 (28.3)
Dairy consumption	Yes	1417/2278 (62.2)	868/1396 (62.2)
	No	861/2278 (37.8)	528/1396 (37.8)

### Age, gender, BMI, education

There was no significant correlation between age and the DHQ total (*r* = 0.043, *P* = 0.052) and no significant difference for gender (*P* = 0.434). Those with a higher level of education were more likely to report healthy dietary habits (*P* < 0.001), while a higher body mass index (BMI) correlated with a lower DHQ total score (*r* = −0.294, *P* < 0.001).

### Health-related quality of life

A moderate, positive correlation was observed between the DHQ total score and the PHC of the MSQOL-54 (*r* = 0.322, *P* < 0.001). For the DHQ total score and the MHC, a lesser positive correlation was observed (*r* = 0.288, *P* < 0.001).

**Figure 1:**
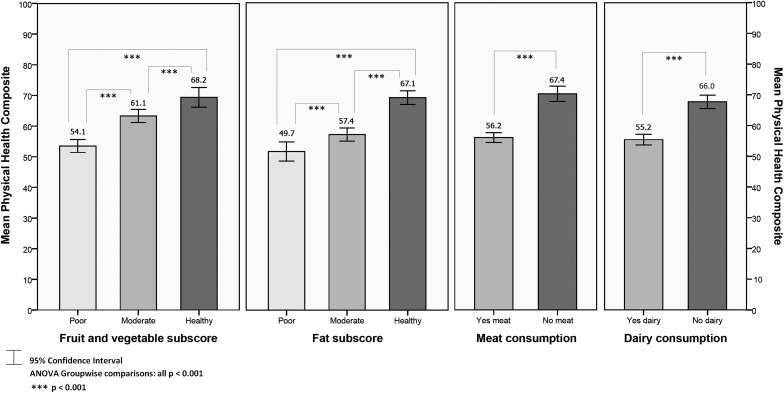
Mean PHC by dietary factors.

**Figure 2:**
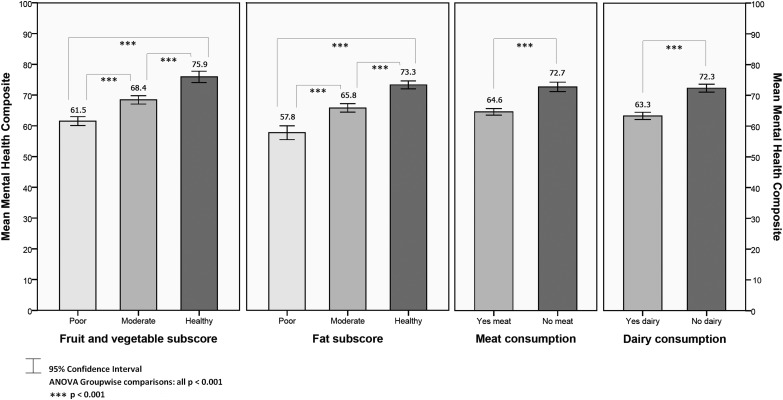
Mean MHC by dietary factors.

Analyses were conducted to explore differences in PHC (Fig. 1) and MHC (Fig. 2) scores between those determined as having ‘poor’, ‘moderate’, or ‘healthy’ diets across the two dietary subscores: fruit and vegetables, and fat. All group and paired differences were statistically significant (*P* < 0.001); those reporting more healthy habits in relation to their intake of fruit and vegetables and fat were significantly more likely to have better HRQOL. Respondents reporting no meat consumption or no dairy consumption had significantly higher PHC and MHC scores (*P* < 0.001), compared to meat and dairy consumers.

In regression analyses, the model for age, gender, and DHQ total score significantly predicted both the PHC and MHC scores, accounting for 15.6 and 7.8% of variability, respectively. After controlling for gender (not a significant covariate) and age (significant for PHC: *B* = −0.484, 95% CI −0.575 to −0.393, *P* < 0.001) for every one-point increase on the DHQ total, the PHC and MHC increased by 0.6 and 0.5, respectively (PHC: *B* = 0.589, 95% CI 0.513–0.666, *P* < 0.001; MHC: *B* = 0.485, 95% CI 0.410–0.560, *P* < 0.001).

Regression analyses for the dietary subgroups revealed the covariates age, healthy fruit and vegetable subscore, and moderate or healthy fat subscore significantly predicted both the PHC and the MHC. Despite meat and dairy having significant associations with better quality of life in bivariate analyses, this was not consistent in the regression models, with only dairy significantly predicting a higher MHC score (Table 4).

**Table 4:** Predicting the PHC and MHCs with dietary subgroups

Dependent	Covariates	*B*	95% CI		*P*	Adjusted *R*^2^
			Lower	Upper		
PHC	Age, years	−0.469	−0.561	−0.377	** < 0.001**	0.150
	Gender, male	1.266	−1.128	3.661	0.300	
	Fruit and vegetable, healthy*	6.431	3.091	9.770	** < 0.001**	
	Fruit and vegetable, moderate*	1.630	−0.730	3.990	0.176	
	Fat, healthy*	11.985	8.376	15.594	** < 0.001**	
	Fat, moderate*	6.883	4.135	9.631	** < 0.001**	
	Meat, non-consumer	2.352	−0.317	5.021	0.084	
	Dairy, non-consumer	2.536	−0.038	5.111	0.053	
MHC	Age, years	0.094	0.007	0.181	**0.034**	0.081
	Gender, male	−0.449	−2.835	1.936	0.712	
	Fruit and vegetable, healthy*	7.599	4.368	10.829	** < 0.001**	
	Fruit and vegetable, moderate*	2.136	−0.112	4.383	0.062	
	Fat, healthy*	8.325	4.861	11.790	** < 0.001**	
	Fat, moderate*	5.463	2.817	8.109	** < 0.001**	
	Meat, non-consumer	0.608	−1.961	3.178	0.642	
	Dairy, non-consumer	3.889	1.442	6.335	**0.002**	

*B* is the unstandardized regression coefficient and 95% CI is the 95% confidence interval of the unstandardized regression coefficient.

*Compared to the ‘poor’ group.

### Disability

Analyses of the level of disability and the DHQ total score indicated a significant difference (*P* < 0.001) in mean DHQ scores across the three disability groups: mild (80.6, 95% CI 79.9–81.3), moderate (77.4, 95% CI 76.6–78.3), and major (76.1, 95% CI 74.6–77.6). *Post-hoc* analyses revealed a small but significant difference in mean DHQ scores between the ‘mild’ and ‘moderate’ disability groups (*P* < 0.001), and the ‘mild’ and ‘major’ disability groups (*P* < 0.001), but not between ‘moderate’ to ‘major’ disability (*P* = 0.296), with those with a lower level of disability having a healthier diet on average.

Analyses of the level of disability among dietary subgroups found significant differences across fruit and vegetables, fat, meat, and dairy (all *P* < 0.001; Table 5). Cross tabulations showed trends towards greater disability among those with poorer subscores, and meat and dairy consumers. Respondents in the mild disability group were more likely to have a healthy fruit and vegetable subscore and healthy fat subscore, and more likely not to eat meat or dairy. Those with moderate disability were more likely to be in the poor fruit and vegetable group, poor or moderate fat group, and among meat and dairy consumers. Results were similar for the major disability group.

**Table 5:** Level of disability and disease activity by dietary subgroups

Dietary subgroups		Level of disability (PDDS)					Disease activity				
		Mild, *n* (%)	Moderate, *n* (%)	Major, *n* (%)	Total, *n* (%)	*P*	Decreasing, *n* (%)	Stable, *n* (%)	Increasing, *n* (%)	Total, *n* (%)	*P*
Fruit and vegetable subscore	Poor	469 (50.0)†	356 (38.0)*	113 (12.0)*	938 (100)	<0.001	212 (39.7)	132 (24.7)	190 (35.6)*	534 (100)	0.004
	Moderate	538 (57.5)	302 (32.3)	95 (10.2)	935 (100)		242 (45.0)	146 (27.1)	150 (27.9)	538 (100)	
	Healthy	225 (63.7)*	106 (30.0)	22 (6.2)†	353 (100)		87 (44.4)	65 (33.2)*	44 (22.4)†	196 (100)	
	Total	1232 (55.3)	764 (34.3)	230 (10.3)	2226 (100)		541 (42.7)	343 (27.1)	384 (30.3)	1268 (100)	
Fat subscore	Poor	188 (48.8)†	156 (40.5)*	41 (10.6)	385 (100)	<0.001	77 (33.9)†	64 (28.2)	86 (37.9)*	227 (100)	<0.001
	Moderate	499 (51.0)†	358 (36.6)*	121 (12.4)*	978 (100)		231 (42.2)	137 (25.0)	180 (32.8)	548 (100)	
	Healthy	510 (65.2)*	218 (27.9)†	54 (6.9)†	782 (100)		216 (47.9)*	129 (28.6)	106 (23.5)†	451 (100)	
	Total	1197 (55.8)	732 (34.1)	216 (10.1)	2145 (100)		524 (42.7)	330 (26.9)	372 (30.3)	1226 (100)	
Meat consumption	Yes	849 (50.8)†	624 (37.4)*	197 (11.8)*	1670 (100)	<0.001	378 (40.6)†	243 (26.1)	309 (33.2)*	930 (100)	0.002
	No	407 (66.7)*	161 (26.4)†	42 (6.9)†	610 (100)		172 (47.1)*	109 (29.9)	84 (23.0)†	365 (100)	
	Total	1256 (55.1)	785 (34.4)	239 (10.5)	2280 (100)		550 (42.5)	352 (27.2)	393 (30.3)	1295 (100)	
Dairy consumption	Yes	717 (51.0)†	531 (37.8)*	158 (11.2)	1406 (100)	<0.001	321 (39.7)†	217 (26.8)	271 (33.5)*	809 (100)	0.003
	No	535 (62.2)*	248 (28.8)†	77 (9.0)	860 (100)		230 (47.5)*	133 (27.5)	121 (25.0)†	484 (100)	
	Total	1252 (55.3)	779 (34.4)	235 (10.4)	2266 (100)		551 (42.6)	350 (27.1)	392 (30.3)	1293 (100)	

*(Bold font) Denotes significantly over-represented as determined by standardized adjusted residuals.

†Denotes significantly under-represented as determined by standardized adjusted residuals.

In ordinal regression modelling, after controlling for gender (not a significant covariate) and age (OR = 1.088, 95% CI 1.078–1.099, *P* < 0.001), for every one-point increase in DHQ total, the odds of having a higher level of disability was associated with a 3.0% reduction (OR = 0.970, 95% CI 0.963–0.977, *P* < 0.001). The odds of having a higher level of disability was significantly lower for those in the healthy and moderate fruit and vegetable groups and the healthy fat group, compared to the poor groups (Table 6). Despite bivariate analyses showing associations with lower disability for those not consuming meat and dairy, this finding did not remain after controlling for covariates in this regression model.

**Table 6:** Predicting a higher level of disability with dietary subgroups

Covariates	Exp(*B*)	95% CI		*P*
		Lower	Upper	
Age, years	1.089	1.078	1.099	** < 0.001**
Gender, male	1.079	0.851	1.368	0.530
Fruit and vegetable, healthy*	0.598	0.430	0.833	**0.002**
Fruit and vegetable, moderate*	0.794	0.636	0.991	**0.042**
Fat, healthy*	0.582	0.412	0.820	**0.002**
Fat, moderate*	0.920	0.711	1.189	0.524
Meat, non-consumer	0.773	0.595	1.006	0.055
Dairy, non-consumer	1.214	0.952	1.548	0.118

Exp(*B*) = Odds ratio (exponential of the log odds).

95% CI is the 95% confidence interval of the odds ratio.

*Compared to the ‘poor’ group.

Nagelkerke pseudo *R*^2^ = 0.212.

### Relapse rate

Among participants with relapsing–remitting MS, there was a small, inverse correlation between the DHQ total score and mean 12 month relapse rate (*r* = −0.117, *n* = 1252, *P* < 0.001); that is a healthier diet was associated with a lower relapse rate, albeit marginal. This finding concurred with regression analysis: after controlling for gender (not a significant covariate) and age (estimate = 0.974, 95% CI 0.965–0.983, *P* < 0.001), for every one-point increase on the DHQ total, the incident rate of relapses over a 12-month period decreased by 1.2% (estimate = 0.988, 95% CI 0.981–0.995, *P* = 0.001).

**Figure 3:**
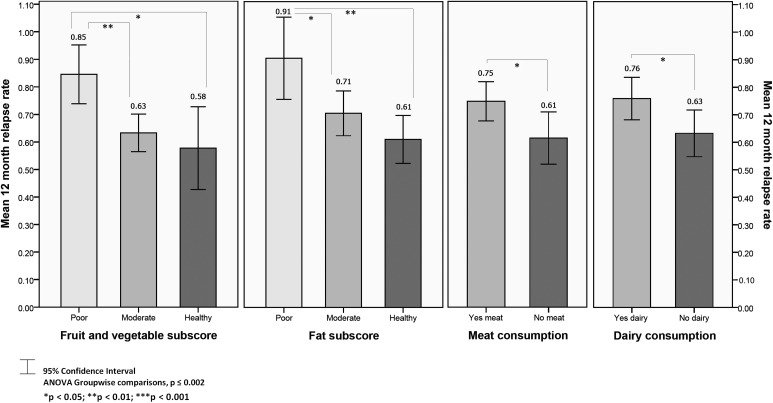
Mean relapse rate by dietary factors.

For the relapsing–remitting sample, there was a significant difference in relapse rate for fruit and vegetable, fat, meat and dairy intake with higher relapse rates for those with poorer subscores, and meat and dairy consumers (all *P* < 0.05; Fig. 3). For fruit and vegetable and fat subscores, *post-hoc* comparisons revealed significant differences in mean relapse rate between poor and healthy (fruit and vegetable *P* = 0.012; fat *P* = 0.001) and poor and moderate groups (fruit and vegetable *P* = 0.003; fat *P* = 0.029); but not between moderate and healthy groups (fruit and vegetable *P* = 0.786; fat *P* = 0.290). Compared to the poor groups, the greatest mean percentage difference in relapse rate observed was for respondents in the healthy fat group; 33.0% lower on average. Respondents reporting meat or dairy consumption were more likely to have a higher mean relapse rate than those not consuming meat (*P* = 0.040) or dairy (*P* = 0.037). These findings were not supported by regression analysis, with age being the only significant predictor of 12-month relapse rate in a regression model with gender, fruit and vegetable subscore, fat subscore, meat and dairy (estimate = 0.974, 95% CI 0.965–0.984, *P* < 0.001).

### Disease activity

Analyses of participants with relapsing–remitting MS indicated a significant difference and trends towards lower mean DHQ total scores across the three disease activity groups: decreasing (80.9, 95% CI 79.9–81.9), stable (79.6, 95% CI 78.2–81.0), and increasing (76.7, 95% CI 75.4–78.0); (*P* < 0.001). *Post-hoc* comparisons showed significant differences in mean DHQ total scores between the decreasing and increasing groups (*P* < 0.001), and stable and increasing groups (*P* = 0.005), but not between stable and decreasing (*P* = 0.319).

Respondents with increasing disease activity were more likely to be in the poor fruit and vegetable and fat groups and consume meat and dairy. Those with stable disease activity were more likely to be in the healthy fruit and vegetable group, while those with decreasing activity were more likely to be in the healthy fat group and not consume meat or dairy (Table 5).

Regression modelling with the DHQ total score was generally poor; the covariates only marginally improved the ability to predict increasing (worsening) disease activity. The DHQ total score had an odds ratio of 0.977 (95% CI 0.967–0.987, *P* < 0.001) after controlling for age (not a significant covariate) and gender (males, OR = 0.618, 95% CI 0.421–0.907, *P* = 0.014). Dietary subgroups did not improve the ability to predict increasing disease activity; only gender was a significant covariate in this model (males, OR 0.619, 95% CI 0.421–0.912, *P* = 0.015).

## Discussion

### Summary of main findings

This study is a unique and comprehensive examination of the dietary habits of a large sample of people with MS and the association of diet with several MS health outcomes. Overall, the study participants appear to have healthy dietary habits, as indicated by their scores on the DHQ total, subscores, and meat and dairy intake (Tables 1 and 3). It is unusual to observe such a large proportion of people who have excluded meat and dairy from their diets, and this is likely a reflection of the recruitment technique employed, which targeted online communities of people with MS actively engaged in lifestyle modification. Participants of the study represent an international sample of people with MS, mostly from English-speaking Western countries, with diverse clinical characteristics including different types of MS.

This study supports strong and significant associations of healthy dietary habits with better physical and mental HRQOL and lower levels of disability. Bivariate analyses show that better quality of life and a lower level of disability are more likely among people with MS consuming a diet with a higher intake of fruit and vegetables, healthy fat intake, and no meat or dairy. Multivariate regression models demonstrate that every 10-point increase on the DHQ total score towards a healthier diet predicts nearly a six-point and five-point increase in physical and mental HRQOL, respectively, and 30% less likelihood of a higher level of disability. These results are both statistically and clinically significant. The tool used to measure HRQOL is derived from the SF-36, for which a five-point change in composite scores is generally considered to be the minimal clinically important difference.^41,42^ The PDDS disability measure correlates highly with the EDDS, and a one-point difference would be considered clinically significant^34^; our groups are collapsed further so that a higher level of disability represents up to a three-point difference.

With a closer examination of dietary factors, fruit and vegetable, and fat intake appear to be important factors in both HRQOL and disability. By contrast, the association between dairy or meat consumption and HRQOL or disability, while suggestive, is inconclusive due to discrepancies between bivariate and multivariate findings. The most pronounced statistically and clinically significant differences were observed for respondents in the healthy fat group. After accounting for the other dietary factors, age and gender, compared to respondents in the poor group, those in the healthy fat group display nearly a 12-point increase in PHC and over an eight-point increase in MHC and a 42% lower likelihood of a higher level of disability. Interestingly, despite the findings in relation to the healthy fat group, the moderate fat group is not a significant predictor of disability. This might suggest that a more rigorous approach to dietary fat is required, which supports the work of Swank where those trial participants adhering rigorously to a very low saturated fat diet with supplementation of PUFAs, had the greatest health gains.^14^ The regression models account for a small-to-moderate amount of variability for the HRQOL and disability outcomes. This suggests that dietary factors are important predictors of these measures, but there remain other factors not accounted for in this model, that require further investigation.

Unlikely HRQOL and disability outcomes, the results for relapse rate and disease activity among people with relapsing–remitting MS are much less robust and require further examination. Despite significant trends in bivariate analyses towards slightly lower relapse rates and stable or decreasing disease activity associated with higher fruit and vegetable intake, healthy fat intake, and an absence of meat and dairy in the diet, these same dietary factors are not significant predictors of relapse rate or disease activity in regression analyses. Interestingly, the total dietary score is significantly associated with relapse rate and disease activity outcomes, but the magnitude of change in both models is small. Furthermore, estimates of the model fit for both outcome variables are poor, suggesting that other variables play a more important role than dietary habits in predicting relapse rate or disease activity.

An in-depth understanding of the biological interplay of nutrition with cell signalling pathways is important to our understanding of whether dietary observations from this study, and others, have biological plausibility, in particular, whether dietary molecules affect the inflammatory and autoimmune processes involved in MS.^43–45^ At a cellular level, nuclear receptors, transcription factors, and enzymes interact with nutrients and regulate gene expression and nutrient metabolism.^43^ In particular, peroxisome proliferator-activated receptors (PPARs) regulate fatty acid metabolism, and play a key role in inflammatory and immunomodulatory pathways.^43^ PPARs have recently proposed to be at the heart of MS pathogenesis.^19^ Saturated fatty acids interfere with the metabolism of precursors of PUFAs, and a disruption to the balance between saturated and PUFAs may compromise the anti-inflammatory and neuroprotective mechanism of PUFAs, including protecting neurons from the cytotoxic action of tumour necrosis factor -alpha.^44^ Further, saturated fats may participate in capillary obstruction, decreased vessel wall elasticity, and cell membrane fluidity.^43,46^ By contrast, dietary phytosterols contained in legumes, nuts, seeds, and other plant foods are thought to have cholesterol-lowering and possible immunomodulatory effects.^47,48^ Fruits and vegetables are likely to confer additional benefits due to the myriad of intrinsic compounds which can affect inflammatory and oxidative pathways. As free radicals are thought to increase demyelination and axonal damage in MS,^49^ it is the antioxidant properties of polyphenols and carotenoids which may be beneficial in restoring an oxidative equilibrium. Moreover, certain polyphenols such as catechins, and quercetin display anti-inflammatory and immunomodulating properties.^43^ The bioactive role of these dietary molecules supports a rationale for increased fruit and vegetable intake and healthy fat intake among people with MS.

Given the findings of this study and others, should clinicians be recommending adoption of a healthy diet to people with MS on the grounds that it is associated with better MS outcomes and indeed might provide additional benefits in the reduction of other chronic disease risk factors?^50,51^ Patients and clinicians might be concerned that adhering to a very healthy diet limits enjoyment from food and creates stress in social situations, yet this study demonstrates that mental HRQOL appears to be much higher among those who do consume a healthy diet. With proper supervision and advice from a medical practitioner and nutritionist, with well-planned meals, recommended intakes of macro- and micronutrients can easily be met. Therefore, further research should seek to explore the potential benefit of dietary modification in people with MS, through the development of novel collaborations between neurologists, molecular biologists, nutritionists, and patients.

### Limitations

Our data were self-reported, hence there may be inaccuracies where respondents were unsure or unable to recall certain information. Verification of participants’ diagnosis of MS and other self-reported data through medical records or clinicians is desirable but not feasible in the context of this large, international sample of online respondents. In general, dietary intake is very complex to assess through survey methodology. Recall bias is common; there is a tendency for respondents to misreport consumption of food types.^52^ The tool used in this study (DHQ) is a fairly crude measure of dietary intake. It aims to provide a snapshot of the dietary habits of an individual for the purpose of dietary education. It was selected for this study because diet was only one aspect being measured and with around 163 items in the broader survey, taking around 30–40 minutes to complete, a brief dietary tool was necessary to reduce respondent burden. Most validated food frequency questionnaires have over 100 items. Alternative assessment options, such as food diaries or 24-hour dietary recall with nutritional analysis were not feasible with a large international, online participant group as this requires participant training or one-on-one interviews. Therefore, we opted for this brief dietary survey, which is not as robust as more detailed dietary assessments. Apart from recall bias, other limitations to the tool are the limited ability to measure portion sizes, whether dietary habits reported were consistent over time, its applicability across ethnic populations and its validation in an Australian cardiac population. Although the scoring system was adhered to for the purpose of this study, some elements, such as the favourable scoring of margarine use (which is highly processed and usually contains hydrogenated fats or trans fats) should be reconsidered. We suggest greater validation of the tool to support its ongoing use as well as an in-depth analysis of the contribution of diet to MS morbidity using a more comprehensive assessment tool.

Our data may have been affected by responder bias. Although data were de-identified, survey participants were not anonymous so it is possible their responses were biased towards reporting more healthy dietary habits. Those with little interest in diet and health may have been less likely to commence the survey in the first place. In general, participants of this study were highly educated and proactive in seeking out information and adopting a wide range of healthy behaviours. It is possible that the findings observed in relation to diet were actually reflective of associations with other confounding lifestyle behaviours. We also cannot exclude reverse associations; that those who have better physical or mental HRQOL or less disability are more likely to engage in healthier dietary habits, although that seems unlikely. It is not clear whether participants were taking any additional vitamin supplements, which might influence the results. Future studies should seek to account for the use of supplementation. The robustness of these findings could be improved by further validating participants’ self-reported diagnosis and health measures, and using a food frequency questionnaire. Planned regression analyses of the interactions of additional lifestyle factors, as well as data collected at follow-up time points, will assist in validating these findings and measure change in dietary habits and MS outcomes over time.

## Conclusion

This study supports a strong and significant association between dietary habits and MS outcomes, in particular quality of life and level of disability. A higher intake of fruit and vegetables and healthy fat intake appear to be important dietary factors, while dairy and meat consumption require further investigation. Further research, including randomized controlled trials of dietary modification for people with MS, is urgently required, notwithstanding the difficulties of conducting such studies. However, clinicians may consider that providing advice to people with MS around the potential importance of a healthy diet is already warranted, given the observations from this study, and others, and general beneficial effects on health.

## Conflict-of-Interest

Professor George Alexander Jelinek receives royalties from his book “Overcoming Multiple Sclerosis: An Evidence-Based Guide to Recovery.” This project was funded by the Bloom Foundation and a donation from Michael Martin.

## Ethics Approval

Ethical approval was granted by St Vincent's Hospital Melbourne Human Research Ethics Committee (LRR 055/12).

## References

[1] van der Mei IA, Simpson S, Stankovich J, Taylor BV Individual and joint action of environmental factors and risk of MS. Neurol Clin 2011;29:233–55. 21439439 10.1016/j.ncl.2010.12.007

[2] Ascherio A, Munger KL Environmental risk factors for multiple sclerosis. Part II: Noninfectious factors. Ann Neurol 2007;61:504–13. 17492755 10.1002/ana.21141

[3] Sawcer S, Hellenthal G, Pirinen M, Spencer CC, Patsopoulos NA, Moutsianas L, *et al.* Genetic risk and a primary role for cell-mediated immune mechanisms in multiple sclerosis. Nature 2011;476:214–9. 21833088 10.1038/nature10251 PMC3182531

[4] Marrie RA Demographic, genetic, and environmental factors that modify disease course. Neurol Clin 2011;29:323–41. 21439444 10.1016/j.ncl.2010.12.004

[5] Multiple Sclerosis International Federation. Atlas of multiple sclerosis database. London: MSIF, 2013 [updated 2013 Oct;cited 2013 Oct 22]. Available from: http://www.atlasofms.org/index.aspx.

[6] Swank RL, Lerstad O, Strom A, Backer J Multiple sclerosis in rural Norway its geographic and occupational incidence in relation to nutrition. New Engl J Med 1952;246:722–8. 14929306 10.1056/NEJM195205082461901

[7] Alter M, Yamour M Multiple sclerosis prevalence and nutritional factors. Trans Am Neurol Assoc 1973;98:253–4. 4784944

[8] Agranoff BW, Goldberg D Diet and the geographical distribution of multiple sclerosis. Lancet 1974;2:1061–6. 4138048 10.1016/s0140-6736(74)92163-1

[9] Esparza ML, Sasaki S, Kesteloot H Nutrition, latitude, and multiple sclerosis mortality: an ecologic study. Am J Epidemiol 1995;142:733–7. 7572944

[10] Ghadirian P, Jain M, Ducic S, Shatenstein B, Morisset R Nutritional factors in the aetiology of multiple sclerosis: a case–control study in Montreal, Canada. Int J Epidemiol 1998;27:845–52. 9839742 10.1093/ije/27.5.845

[11] Lauer K The risk of multiple sclerosis in the USA in relation to sociogeographic features: a factor-analytic study. J Clin Epidemiol 1994;47:43–8. 8283194 10.1016/0895-4356(94)90032-9

[12] Gusev E, Boiko A, Lauer K, Riise T, Deomina T Environmental risk factors in MS: a case–control study in Moscow. Acta Neurol Scand 1996;94:386–94. 9017026 10.1111/j.1600-0404.1996.tb00050.x

[13] Zhang SM, Willett WC, Hernan MA, Olek MJ, Ascherio A Dietary fat in relation to risk of multiple sclerosis among two large cohorts of women. Am J Epidemiol 2000;152:1056–64. 11117615 10.1093/aje/152.11.1056

[14] Swank RL, Dugan BB Effect of low saturated fat diet in early and late cases of multiple sclerosis. Lancet 1990;336:37–9. 1973220 10.1016/0140-6736(90)91533-g

[15] Weinstock-Guttman B, Baier M, Park Y, Feichter J, Lee-Kwen P, Gallagher E, *et al.* Low fat dietary intervention with omega-3 fatty acid supplementation in multiple sclerosis patients. Prostaglandins Leukot Essent Fatty Acids 2005;73:397–404. 16099630 10.1016/j.plefa.2005.05.024

[16] Marrie RA, Rudick R, Horwitz R, Cutter G, Tyry T, Campagnolo D, *et al.* Vascular comorbidity is associated with more rapid disability progression in multiple sclerosis. Neurology 2010;74:1041–7. 20350978 10.1212/WNL.0b013e3181d6b125 PMC2848107

[17] Weinstock-Guttman B, Zivadinov R, Mahfooz N, Carl E, Drake A, Schneider J, *et al.* Serum lipid profiles are associated with disability and MRI outcomes in multiple sclerosis. J Neuroinflammation 2011;8:127. 21970791 10.1186/1742-2094-8-127 PMC3228782

[18] Weinstock-Guttman B, Zivadinov R, Horakova D, Havrdova E, Qu J, Shyh G, *et al.* Lipid profiles are associated with lesion formation over 24 months in interferon-beta treated patients following the first demyelinating event. J Neurol Neurosurg Psychiatry 2013;84:1186–91. 23595944 10.1136/jnnp-2012-304740

[19] Corthals AP Multiple sclerosis is not a disease of the immune system. Q Rev Biol 2011;86:287–321. 22384749 10.1086/662453

[20] Kennel De March A, De Bouwerie M, Kolopp-Sarda MN, Faure GC, Bene MC, Bernard CC Anti-myelin oligodendrocyte glycoprotein B-cell responses in multiple sclerosis. J Neuroimmunol 2003;135:117–25. 12576231 10.1016/s0165-5728(02)00434-4

[21] Guggenmos J, Schubart AS, Ogg S, Andersson M, Olsson T, Mather IH Antibody cross-reactivity between myelin oligodendrocyte glycoprotein and the milk protein butyrophilin in multiple sclerosis. J Immunol 2004;172:661–8. 14688379 10.4049/jimmunol.172.1.661

[22] Stefferl A, Schubart A, Storch M, Amini A, Mather I, Lassmann H, *et al.* Butyrophilin, a milk protein, modulates the encephalitogenic T cell response to myelin oligodendrocyte glycoprotein in experimental autoimmune encephalomyelitis. J Immunol 2000;165:2859–65. 10946319 10.4049/jimmunol.165.5.2859

[23] Malosse D, Perron H, Sasco A, Seigneurin JM Correlation between milk and dairy product consumption and multiple sclerosis prevalence: a worldwide study. Neuroepidemiology 1992;11:304–12. 1291895 10.1159/000110946

[24] Malosse D, Perron H Correlation analysis between bovine populations, other farm animals, house pets, and multiple sclerosis prevalence. Neuroepidemiology 1993;12:15–27. 8327019 10.1159/000110295

[25] Gilgun-Sherki Y, Melamed E, Offen D The role of oxidative stress in the pathogenesis of multiple sclerosis: the need for effective antioxidant therapy. J Neurol 2004;251:261–8. 15015004 10.1007/s00415-004-0348-9

[26] Zhang SM, Hernan MA, Olek MJ, Spiegelman D, Willett WC, Ascherio A Intakes of carotenoids, vitamin C, and vitamin E and MS risk among two large cohorts of women. Neurology 2001;57:75–80. 11445631 10.1212/wnl.57.1.75

[27] Manouchehrinia A, Constantinescu CS Cost-effectiveness of disease-modifying therapies in multiple sclerosis. Curr Neurol Neurosci Rep 2012;12:592–600. 22782520 10.1007/s11910-012-0291-6

[28] Farinotti M, Vacchi L, Simi S, Di Pietrantonj C, Brait L, Filippini G Dietary interventions for multiple sclerosis. Cochrane Database Syst Rev 2012;12:CD004192. 10.1002/14651858.CD004192.pub3 23235605

[29] Schwarz S, Knorr C, Geiger H, Flachenecker P Complementary and alternative medicine for multiple sclerosis. Mult Scler 2008;14:1113–9. 18632773 10.1177/1352458508092808

[30] Leong EM, Semple SJ, Angley M, Siebert W, Petkov J, McKinnon RA Complementary and alternative medicines and dietary interventions in multiple sclerosis: what is being used in South Australia and why? Complement Ther Med 2009;17:216–23. 19632549 10.1016/j.ctim.2009.03.001

[31] Hadgkiss EJ, Jelinek GA, Weiland TJ, Pereira NG, Marck CH, van der Meer DM Methodology of an international study of people with multiple sclerosis recruited through web 2.0 platforms: demographics, lifestyle, and disease characteristics. Neurol Res Int 2013;2013:580596. 23691313 10.1155/2013/580596 PMC3649686

[32] Vickrey BG, Hays RD, Harooni R, Myers LW, Ellison GW A health-related quality of life measure for multiple sclerosis. Qual Life Res 1995;4:187–206. 7613530 10.1007/BF02260859

[33] Hohol MJ, Orav EJ, Weiner HL Disease steps in multiple sclerosis: a simple approach to evaluate disease progression. Neurology 1995;45:251–5. 7854521 10.1212/wnl.45.2.251

[34] Hohol MJ, Orav EJ, Weiner HL Disease steps in multiple sclerosis: a longitudinal study comparing disease steps and EDSS to evaluate disease progression. Mult Scler 1999;5:349. 10516779 10.1177/135245859900500508

[35] Learmonth YC, Motl RW, Sandroff BM, Pula JH, Cadavid D Validation of patient determined disease steps (PDDS) scale scores in persons with multiple sclerosis. BMC Neurol 2013;13:37. 23617555 10.1186/1471-2377-13-37 PMC3651716

[36] McKellar S, Horsley P, Chambers R, Pullen M, Vandersee P, Clarke C, *et al.* Development of the Diet Habits Questionnaire for use in cardiac rehabilitation. Aust J Prim Health 2008;14:43–7.

[37] Dobson AJ, Blijlevens R, Alexander HM, Croce N, Heller RF, Higginbotham N, *et al.* Short fat questionnaire: a self-administered measure of fat-intake behaviour. Aust J Pub Health 1993;17:144–9. 8399708 10.1111/j.1753-6405.1993.tb00123.x

[38] Wright JL, Scott JA The fat and fibre barometer, a short food behaviour questionnaire: reliability, relative validity and utility. Aust J Nutr Diet 2000;57:33–9.

[39] Jelinek GA, Hadgkiss EJ, Weiland TJ, Pereira NG, Marck CH, van der Meer DM Association of fish consumption and omega 3 supplementation with quality of life, disability and disease activity in an international cohort of people with multiple sclerosis. Int J Neurosci 2013;123:792–801. 23713615 10.3109/00207454.2013.803104 PMC3821380

[40] Weiland TJ, Hadgkiss EJ, Jelinek GA, Pereira NG, Marck CH, van der Meer DM The association of alcohol consumption and smoking with quality of life, disability and disease activity in an international sample of people with multiple sclerosis. J Neurol Sci 2014;336:211–9. 24290614 10.1016/j.jns.2013.10.046

[41] Kappos L, Gold R, Arnold DL, Bar-Or A, Giovannoni G, Selmaj K, *et al.* Quality of life outcomes with BG-12 (dimethyl fumarate) in patients with relapsing-remitting multiple sclerosis: The DEFINE study. Mult Scler 2014;20:243–52. 24150779 10.1177/1352458513507817

[42] Norman GR, Sloan JA, Wyrwich KW Interpretation of changes in health-related quality of life: the remarkable universality of half a standard deviation. Med Care 2003;41:582–92. 12719681 10.1097/01.MLR.0000062554.74615.4C

[43] Riccio P, Rossano R, Liuzzi GM May diet and dietary supplements improve the wellness of multiple sclerosis patients? A molecular approach. Autoimmune Dis 2011;2010:249842. 21461338 10.4061/2010/249842 PMC3065662

[44] Das UN Is there a role for saturated and long-chain fatty acids in multiple sclerosis? Nutrition 2003;19:163–6. 12591552 10.1016/s0899-9007(02)00923-1

[45] Schwarz S, Leweling H Multiple sclerosis and nutrition. Mult Scler 2005;11:24–32. 15732263 10.1191/1352458505ms1119oa

[46] Swank RL, Goodwin JW How saturated fats may be a causative factor in multiple sclerosis and other diseases. Nutrition 2003;19:478. 12714108 10.1016/s0899-9007(02)01099-7

[47] Valerio M, Liu HB, Heffner R, Zivadinov R, Ramanathan M, Weinstock-Guttman B, *et al.* Phytosterols ameliorate clinical manifestations and inflammation in experimental autoimmune encephalomyelitis. Inflamm Res 2011;60:457–65. 21136279 10.1007/s00011-010-0288-z

[48] Desai F, Ramanathan M, Fink CS, Wilding GE, Weinstock-Guttman B, Awad AB Comparison of the immunomodulatory effects of the plant sterol beta-sitosterol to simvastatin in peripheral blood cells from multiple sclerosis patients. Int Immunopharmacol 2009;9:153–7. 19022404 10.1016/j.intimp.2008.10.019

[49] von Geldern G, Mowry EM The influence of nutritional factors on the prognosis of multiple sclerosis. Nat Rev Neurol 2012;8:678–89. 23026980 10.1038/nrneurol.2012.194

[50] Rosell M, Appleby P, Spencer E, Key T Weight gain over 5 years in 21,966 meat-eating, fish-eating, vegetarian, and vegan men and women in EPIC-Oxford. Int J Obes (Lond) 2006;30:1389–96. 16534521 10.1038/sj.ijo.0803305

[51] Appleby PN, Davey GK, Key TJ Hypertension and blood pressure among meat eaters, fish eaters, vegetarians and vegans in EPIC-Oxford. Public Health Nutr 2002;5:645–54. 12372158 10.1079/PHN2002332

[52] Coulston AM, Boushey CJ, Ferruzzi MG Dietary assessment methodology. Nutrition in the prevention and treatment of disease. San Diego: Academic Press; 2013, chapter 1.

